# The use of feed-grade amino acids in lactating sow diets

**DOI:** 10.1186/s40104-017-0223-z

**Published:** 2018-01-12

**Authors:** Laura Greiner, Pairat Srichana, James L. Usry, Casey Neill, Gary L. Allee, Joseph Connor, Kevin J. Touchette, Christopher D. Knight

**Affiliations:** 1 Carthage Innovative Sow Solutions, LLC, Carthage, IL 62321 USA; 2 Department of Animal Science, University of Missouri-Columbia, Columbia, MO 65211 USA; 3 Present address: Charoen Pokphand Group Co., Ltd., 14th Floor, 313 Silom Rd., Bangrak, Bangkok, 10500 Thailand; 4 Ajinomoto Heartland, Inc., Chicago, IL 60631 USA; 5 Present address: Micronutrients, 1550 Research Way, Indianapolis, IN 46231 USA; 6 PIC, Hendersonville, TN 37075 USA; 7 Present address:, Pipestone, USA; 8 Novus International, Inc., St. Charles, MO 63304 USA

**Keywords:** Amino acid ratio, Lactation, *L*-lysine, Sow

## Abstract

**Background:**

The use of feed grade amino acids can reduce the cost of lactation feed. With changing genetics, increasing feed costs, and higher number of pigs weaned with heavier wean weights further evaluation of higher inclusion levels of feed-grade amino acid in lactation diets than previously published is warranted. Two experiments (Exp.) were conducted to determine the optimal inclusion level of *L*-lysine HCl to be included in swine lactation diets while digestible lysine levels remain constant across dietary treatments and allowing feed grade amino acids to be added to the diet to maintain dietary ratios relative to lysine to maximize litter growth rate and sow reproductive performance. Furthermore, the studies were to evaluate minimal amino acid ratios relative to lysine that allows for optimal litter growth rate and sow reproductive performance.

**Results:**

Exp. 1: Increasing *L*-lysine HCl resulted in similar gilt feed intake, litter, and reproductive performance. Average litter gain from birth to weaning was 2.51, 2.49, 2.59, 2.43, and 2.65 kg/d when gilts were fed 0.00, 0.075, 0.150, 0.225, and 0.30% *L*-lysine HCl, respectively. Exp. 2: The average litter gain from birth to weaning was 2.68, 2.73, 2.67, 2.70, and 2.64 kg/d (*P* < 0.70) when sows were fed 0.1, 0.2, 0.3, 0.4, and 0.4% *L*-lysine HCl plus valine, respectively. No other differences among dietary treatments were observed.

**Conclusions:**

Collectively, these studies demonstrate corn-soybean meal based lactation diets formulated with a constant SID lysine content for all parities containing up to 0.40% *L*-lysine HCl with only supplemental feed grade threonine and a methionine source have no detrimental effect on litter growth rate and subsequent total born.

## Background

Feed-grade amino acids have been extensively used as alternatives to intact protein in grow-finish diets to maintain animal performance at an optimized cost and to reduce nitrogen excretion. The use of feed grade amino acids as a replacement for intact proteins can result in a current savings of 1-2.5 US dollars per sow per lactation period. However, data are limited as to how much feed-grade amino acids can be used in sow lactation diets to replace intact protein without reducing sow lactation and reproductive performance. Furthermore, the data evaluating the use of feed-grade amino acids has been conflicting in the results. For example, the addition of 0.15% *L*-lysine HCl to 13% crude protein diets has been shown to improve milk production in sows but further addition failed to improve performance suggesting one or more amino acids other than lysine are limiting [1]. A separate study demonstrated that adding more than 0.075% *L*-lysine HCl to diets of lactating sows that are losing weight in a commercial setting can increase pre-weaning mortality and decreased the number of pigs weaned [2]. However, a study did demonstrate that when high levels of feed-grade amino acids (0.58% to 0.73% *L*-lysine HCl) were fed during lactation, lactation performance was equal to conventional diets, which suggests that feed-grade amino acids can be added to the lactation diets without impacting sow or litter performance [3]. Other studies have evaluated other feed-grade amino acids outside of *L*-lysine HCl. One study evaluated *L*-threonine by feeding 0.37% *L*-lysine HCl, which resulted in increased feed intake and reduced sow weight loss [4]. The purpose of these studies was to evaluate the ability to use feed grade amino acids in sow lactation diets and to determine the acceptable feed grade amino acid inclusion levels of the first limiting amino acid. Therefore, the objective of these studies was to determine the optimal inclusion level of *L*-lysine HCl to be included in swine lactation diets while digestible lysine levels remain constant across dietary treatments and allowing feed grade amino acids to be added to the diet to maintain dietary ratios relative to lysine to maximize litter growth rate and sow reproductive performance. Furthermore, the studies were to evaluate minimal amino acid ratios relative to lysine that allows for optimal litter growth rate and sow reproductive performance.

## Methods

Both studies were conducted in a commercial 6,000 sow farm located in Western Illinois of the United States. All animal care practices were conducted by following routine farm management procedures and Pork Quality Assurance (PQA) guidelines [5]; therefore, the experimental procedures were not submitted for approval to an Animal Care Committee.

### Exp. 1

From November 2005 through February 2006, two hundred and fifty-nine primiparous gilts (PIC USA, Camborough 22) were used to determine the effect of feed-grade amino acid level [*L*-lysine HCl, *L*-threonine, and Alimet® (88% 2-hydroxy, 4-methylthio butanoic acid, HMTBa, Novus International, St. Charles, MO)] of the diet on lactation performance. Gilts (195.99 ± 21.38 kg; mean ± standard deviation) were randomly allotted to one of five experimental diets at approximately 112 d of gestation and were fed those diets for the duration of the lactation period (19.3 ± 1.6 d).

Dietary treatments were formulated to contain different levels of *L*-lysine HCl (0.00, 0.075, 0.150, 0.225, and 0.300%; diet 1, 2, 3, 4, and 5 respectively), replacing intact lysine, mainly from soybean meal. Feed-grade threonine (Thr) and sulfur amino acids ratios relative to lysine (Lys) (on a digestible basis) were supplemented to have the same ratios as in the corn-soybean meal control diet (Table 1). All diets were formulated to have the same total lysine (1.20%) and 3.45 Mcal ME/kg and contained vitamins and minerals that exceeded NRC recommendations [6]. Metabolizable energy (ME) values for individual ingredients were from the NRC [6].

**Table 1:** Diet composition of lactation diets varying in *L*-lysine HCl, Exp. 1 (as-fed)

	Diet				
Ingredient, %	1	2	3	4	5
Corn	57.49	59.20	61.49	63.29	65.44
Soybean meal, 46%	34.71	32.77	30.44	28.51	26.18
Choice White Grease	4.00	4.00	4.00	4.00	4.00
Monocalcium phosphate 21%	1.70	1.70	1.70	1.70	1.75
Limestone	1.25	1.25	1.25	1.25	1.25
Salt	0.50	0.50	0.50	0.50	0.50
Vitamin/trace mineral/phytase^a^	0.25	0.25	0.25	0.25	0.25
Choline chloride	0.10	0.10	0.10	0.10	0.10
*L*-lysine HCl	0.000	0.075	0.150	0.225	0.300
*L*-threonine	0.000	0.035	0.0675	0.103	0.135
HMTBa^b^	0.000	0.025	0.050	0.075	0.100
Calculated Nutrients Composition					
ME, Mcal/kg^c^	3.450	3.452	3.454	3.456	3.456
Crude protein, %	21.0	20.4	19.6	18.9	18.1
SID^d^ Lys, %	1.06	1.07	1.07	1.08	1.08
SID M + C^e^, %	0.62	0.62	0.62	0.63	0.63
SID Thr, %	0.70	0.71	0.71	0.71	0.71
SID Trp, %	0.23	0.22	0.21	0.19	0.18
SID Val, %	0.89	0.86	0.81	0.79	0.75
g SID Lys/Mcal NRC ME	3.07	3.09	3.09	3.12	3.12
SID M + C:Lys	0.58	0.58	0.58	0.58	0.58
SID Thr:Lys	0.66	0.66	0.66	0.66	0.66
SID Trp:Lys	0.22	0.21	0.20	0.18	0.17
SID Val:Lys	0.84	0.80	0.76	0.73	0.69

^a^Premix supplied per kilogram of diet: vitamin A, 11,000 IU; vitamin D_3_, 1760 IU; vitamin E (*DL*-alpha tocopheryl acetate), 66 IU; vitamin K (menadione activity), 3.96 mg; riboflavin, 8.25 mg; *D*-pantothenic acid, 29.70 mg; niacin, 49.5 mg; vitamin B_12_, 0.04 mg; *D*-Biotin, 0.22 mg; folic acid, 1.65 mg; pyridoxine, 5.1 mg; Zn (ZnSO_4_), 170 mg; Cu (CuSO_4_), 20 mg; Fe (FeSO_4_), 170 mg; Mn (MnSO_4_), 50 mg; I (ethylenediamine dihydriodide), 0.35 mg; and Se (Na_2_SeO_3_), 0.30 mg. Phytase was provided as Optiphos (Huevepharma, Sofia, Bulgaria) and added 750 phytase units/kg diet

^b^ALIMET is a trademark of Novus International, Inc., and is registered in the United States and other countries. Methionine source. 88% 2-hydroxy, 4-methylthio butanoic acid, HMTBa

^c^Calculated from NRC (1998)

^d^SID = Standardized ileal digestible

^e^M + C = Methionine + cysteine

### Exp. 2

This study was conducted August 2010 through December 2010. Five hundred and forty-five primiparous and multiparous sows (parity 1 through 3, PIC Cambourough 29) were blocked by parity and randomly allotted to one of five dietary treatments at approximately 112 d of gestation and were fed the experimental diet for the duration of the lactation period (20.6 ± 1.1 d).

Five diets used in this experiment contained different levels of *L*-lysine HCl (0.1, 0.2, 0.3, 0.4, and 0.4% + valine (Val); diet 1, 2, 3, 4, and 5 respectively), replacing intact lysine, mainly from soybean meal (Table 2). *L*-threonine and HMTBa were included in the diets as necessary in order to maintain the standardized ileal digestibile (SID) Thr:Lys and SID methionine + cysteine (M + C):Lys ratios above 65% and 49%, respectively. In addition, dietary treatment 5 was supplemented with 0.025% valine to increase the SID Val:Lys ratio from 64 in diet 4 to 69, respectively. All diets were formulated to have the same SID lysine (1.12%) and 3.45 Mcal ME/kg and contained vitamins and minerals that exceeded NRC recommendations [6]. Energy values for individual ingredients were calculated using the ME values from the NRC [6].

**Table 2:** Diet composition of lactation diets varying in *L*-lysine HCl, Exp. 2 (as-fed)

	Diet				
Ingredient, %	1	2	3	4	5
Corn	58.00	61.01	63.97	66.94	66.89
Soybean meal, 46%	34.00	38.05	27.75	24.60	24.60
Choice White Grease	4.00	4.00	4.00	4.00	4.00
Monocalcium phosphate 21%	1.65	1.65	1.65	1.65	1.65
Limestone	1.35	1.35	1.35	1.35	1.35
Salt	0.50	0.50	0.50	0.50	0.50
Vitamin/trace mineral/phytase^a^	0.25	0.25	0.25	0.25	0.25
Choline chloride	0.10	0.10	0.10	0.10	0.10
*L*-lysine HCl	0.10	0.20	0.30	0.40	0.40
*L*-threonine	0.04	0.80	0.20	0.18	0.18
HMTBa^b^	0.00	0.00	0.00	0.04	0.04
*L*-valine	0.00	0.00	0.00	0.00	0.06
Calculated Nutrients Composition					
NRC ME, Mcal/kg^c^	3.450	3.450	3.450	3.450	3.450
Crude protein, %	21.09	19.97	18.87	17.76	16.72
SID^d^ Lys, %	1.12	1.12	1.12	1.12	1.12
SID M + C^e^, %	0.62	0.58	0.55	0.55	0.55
SID Thr, %	0.73	0.73	0.73	0.73	0.73
SID Trp, %	0.22	0.21	0.19	0.18	0.18
SID Val, %	0.87	0.83	0.77	0.72	0.77
g SID Lys/Mcal NRC ME	3.24	3.24	3.24	3.24	3.24
SID M + C:Lys	0.55	0.52	0.49	0.49	0.49
SID Thr:Lys	0.65	0.65	0.65	0.65	0.65
SID Trp:Lys	0.20	0.19	0.17	0.16	0.16
SID Val:Lys	0.78	0.74	0.69	0.64	0.69

^a^Premix supplied per kilogram of diet: vitamin A, 11,000 IU; vitamin D_3_, 1,760 IU; vitamin E (*DL-*alpha tocopheryl acetate), 66 IU; vitamin K (menadione activity), 3.96 mg; riboflavin, 8.25 mg; *D*-pantothenic acid, 29.70 mg; niacin, 49.5 mg; vitamin B_12_, 0.04 mg; *D*-Biotin, 0.22 mg; folic acid, 1.65 mg; pyridoxine, 5.1 mg; Zn (ZnSO_4_), 170 mg; Cu (CuSO_4_), 20 mg; Fe (FeSO_4_), 170 mg; Mn (MnSO_4_), 50 mg; I (ethylenediamine dihydriodide), 0.35 mg; and Se (Na_2_SeO_3_), 0.30 mg. Phytase was provided as Optiphos (Huevepharma, Sofia, Bulgaria) and added 750 phytase units/kg diet

^b^ ALIMET is a trademark of Novus International, Inc., and is registered in the United States and other countries. Methionine source. 88% 2-hydroxy, 4-methylthio butanoic acid, HMTBa

^c^Calculated from NRC (1998)

^d^*SID* Standardized ileal digestible

^e^*M + C* Methionine + cysteine

### Lactation feeding

Upon entering the farrowing rooms and prior to farrowing, gilts were fed 1.8 kg/d of the respective lactation diet. After farrowing, gilts were fed 1.8 kg on d 1, 2.7 kg on d 2, 3.6 kg on d 3, and then allowed ad libitum access to feed in Exp. 1. In Exp. 2, prior to farrowing, sows were fed 1.8 kg/d of their respective lactation treatment. After farrowing, sows were fed 1.8 kg on d 1, 2.7 kg on d 2, 3.6 kg on d 3. After d 3, all females were allowed a maximum feed intake per day of 5.2, 5.5, 5.7 kg, respectively.

Feed was delivered to each sow through the automated Howema Feed System (Big Dutchman, Vechta, Germany). In Exp. 1, the dietary treatments 1 and 5 were manufactured at a local feed mill and delivered to the facility. The feed system on site blended dietary treatments 2, 3, and 4 by delivering a set percentage of each diet into a mixing hopper and recording the actual weight of each diet as it was added to the hopper and then proceeded to mix the diet for 4 s prior to delivering each batch of feed to the corresponding sow. In Exp. 2, dietary treatments 1, 4, and 5 were manufactured at a local feed mill and delivery to the facility. At the time of delivery, the system recorded the amount of feed delivered and tracked total lactation consumption per sow. Feed was delivered to each sow via a cable system and was held in a 6.8 kg plastic hopper (Automated Production Systems, Assumption, IL) attached to an InTak feeder (Automated Production Systems, Assumption, IL). Sows had ad libitum access to water throughout lactation.

### Animal husbandry

Sows were moved into the farrowing house at 112 ± 2 d of gestation length and allocated to the experimental diets upon entry into the farrowing house and were fed the allotted dietary treatmentss from the time of entry into the farrowing house until weaning. Sows were housed in conventional farrowing stalls in an environmentally regulated commercial farrow to wean facility (18 to 24 °C) with lights on from 0600 to 1500 h. Sows were housed in a standard farrowing stall with a total dimension of 1.5 m × 2.1 m. Sows farrowed at on average at 115 d of gestation and piglets were cross-fostered within dietary treatment within 24 h of birth. In addition, the number of piglets per sow was equalized across all dietary treatment groups. Tails of piglets were clipped and 200 mg of iron dextran was injected at 3 d of age. Male piglets were surgically castrated on d 3. Pigs were not offered creep feed during the study, but did have access to water. In addition, rubber mats and heat lamps were provided as a source of supplemental heat to the piglets.

### Gilt/sow and litter criteria

The gilts were negative for porcine reproductive and respiratory disease, porcine epidemic diarrhea virus, and transmissible gastroenteritis negative and *Mycoplasma hyopneumoniae* stable. All females were weighed at the time of entry into the farrowing house (Tru-Test, Mineral Wells, TX and J&H Automation, Gridley, IL) and again at the time of weaning. Gilt/sow 48 h post-farrow body weight was determined using the prediction equation: post-farrow weight = [(112 d gestation weight, lbs./2.2) × 0.98]- 20.81 (*R*^2^ = 0.93) (Greiner, unpublished data). In addition, piglet litter weights were recorded at 48 h of age and at weaning (Tru-Test, Mineral Wells, TX and J&H Automation, Gridley, IL). Any mortalities and morbidities were documented by lactation day, sow identification, and removal weight when the pigs were removed from trial. The removal weights and nursing days were calculated back into litter growth rate [(total litter wean weight – total starting litter weight + mortality weights)/((number of pigs weaned × lactation length) + days moralities nursed)]. After weaning, sows were fed ad libitum a conventional gestation diet containing 3.17 Mcal ME/kg and 0.61% total lysine. Sows were checked daily for signs of estrus using a mature boar beginning d 3 after weaning. Estrus was recorded when sows stood to be mounted by a boar, and days from weaning to estrus were also recorded. In addition, the number of sows bred within 10 d of weaning was recorded. The gestation feed was consumed at approximately 3.6 kg/d from weaning to mating and then after mating, feeding levels were adjusted for body condition based on a farm specific feeding scale that allowed body condition to be maintained during the remainder of the gestation period through the use of a visual body condition scoring system. Weaning to mating interval, farrow to subsequent farrow interval and second litter number total born, born alive, stillborns, and mummies were recorded. For second parity litter characteristics, only sows mated within 21 d post-weaning and farrowing as a result of first mating were used.

### Diet analysis

Diets were submitted to Ajinomoto Heartland, Inc. (Chicago, IL) for amino acid analyses following AOAC methodology [7] (Tables 3 and 4).

**Table 3:** Analyzed total amino acid profile for diets used in Exp. 1 on as as-fed basis^a^

		Dietary Treatment^b^				
Total Amino Acid		1	2	3	4	5
Lysine, %	Analyzed	1.18	1.18	1.20	1.21	1.20
	Calculated	1.16	1.15	1.15	1.15	1.16
Methionine + Cysteine, %	Analyzed^c^	0.69	0.69	0.67	0.67	0.66
	Calculated	0.71	0.69	0.67	0.65	0.62
Threonine, %	Analyzed	0.81	0.81	0.81	0.82	0.83
	Calculated	0.79	0.76	0.76	0.77	0.77
Tryptophan, %	Analyzed	0.25	0.24	0.23	0.22	0.21
	Calculated	0.27	0.25	0.24	0.23	0.22
Valine, %	Analyzed	1.01	0.98	0.95	0.95	0.90
	Calculated	0.94	0.90	0.86	0.82	0.77

^a^ Diets were analyzed using HPLC at Ajinomoto Heartland Lab, Chicago, IL

^b^ Dietary treatments were as follows: 1 – 0.00, 2 – 0.075, 3 – 0.150, 4 – 0.225, 5 – 0.300% *L*-lysine HCl inclusion

^c^HMTBa not detected in analysis as it does not contain nitrogen. ALIMET is a trademark of Novus International, Inc., and is registered in the United States and other countries. Methionine source. 88% 2-hydroxy, 4-methylthio butanoic acid, HMTBa

**Table 4:** Analyzed total amino acid profile diets used in Exp. 2 on an as-fed basis^a^

		Dietary Treatment^b^				
Total Amino Acid		1	2	3	4	5
Lys, %	Calculated	1.26	1.25	1.24	1.23	1.23
	Analyzed	1.22	1.31	1.29	1.27	1.28
Met+Cys:Lys	Calculated	0.55	0.53	0.50	0.50	0.50
	Analyzed^c^	0.50	0.49	0.47	0.46	0.45
Thr:Lys, %	Calculated	0.67	0.67	0.67	0.67	0.67
	Analyzed	0.69	0.68	0.65	0.66	0.65
Trp:Lys, %	Calculated	0.20	0.19	0.18	0.16	0.16
	Analyzed	0.22	0.20	0.19	0.17	0.17
Val:Lys, %	Calculated	0.79	0.75	0.71	0.67	0.71
	Analyzed	0.75	0.79	0.75	0.74	0.75

^a^Diets were analyzed using HPLC at Ajinomoto Heartland Lab, Chicago, IL

^b^ Dietary treatments were as follows: 1 – 0.10, 2 – 0.20, 3 – 0.30, 4 – 0.40% *L*-lysine HCl, 5 – 0.40% *L*-lysine HCl inclusion + feed grade valine

^c^HMTBa not detected in analysis as it does not contain nitrogen. ALIMET is a trademark of Novus International, Inc., and is registered in the United States and other countries. Methionine source. 88% 2-hydroxy, 4-methylthio butanoic acid, HMTBa

### Statistical analysis

Data were analyzed by ANOVA using PROC MIXED procedures of SAS (SAS Inst, Inc., Cary, NC) and reported as LS means. In addition, PROC GLIMMIX was used for evaluation of pre-wean morbidity/mortality and percent bred. In Exp. 1, the statistical model included dietary treatment and covariates of the number of pigs per sow and days of lactation. In Exp. 2, the statistical model included dietary treatment, parity (block) and dietary treatment × parity interactions. The sow was the experimental unit. Polynomial coefficients were used to determine linear and quadratic effects on increasing feed-grade lysine inclusion in Exp. 1. In Exp. 2, the polynomial coefficients were used to determine the linear effects among the increasing levels of feed-grade lysine (dietary treatments 1-4) and a then a comparison was conducted to evaluate the statistical difference between dietary treatment 4 and 5 (inclusion of supplemental valine). A value of *P* < 0.05 was considered significant.

## Results

### Exp. 1

Twenty-four gilts did not complete the study. Reasons for removal from the study included: illness resulting in significant weight loss or feed refusal, death, failure to wean more than 7 pigs, or a reduced lactation period due to an early weaning event. The average lactation length in this study was 19.3 ± 1.6 d. Increasing the *L*-lysine HCl inclusion decreased crude protein (CP) content of the diets from 21.0 to 18.2%. Increasing *L*-lysine HCl resulted in similar litter and reproductive performance compared with the control diet (Table 5). The inclusion of *L*-lysine HCl did not alter average daily feed intake (ADFI) which averaged 5.99 ± 0.93 kg/d. The ME intake and grams of total lysine per day were similar among the five dietary *L*-lysine HCl inclusions. In addition, the inclusion of *L*-lysine HCl did not significantly alter gilt body weight gain (BW), wean-to-estrus interval (WEI), or percent of gilts re-bred in 10 d. The average WEI was 5.5 ± 1.9 d and the percent of females bred within 10 d was 97.9 ± 14.5%. The average litter gain, piglet average daily gain (ADG), and litter mortality were not different between diets when gilts were fed increasing levels of *L*-lysine HCl.

**Table 5:** Evaluation of *L*-lysine HCl on gilt and litter performance for Exp. 1

	Diet^a^								
	1	2	3	4	5	SEM	Trt^b^	Linear	Quadratic
Gilt									
Sample size, *n*	46	49	47	47	46				
Estimated SID Lys, g/d	62.9	64.1	64.3	62.6	66.7				
ADFI, kg	5.88	5.99	6.01	5.85	6.23	0.14	0.33	0.20	0.50
Weight change, %	1.09	1.94	−0.28	2.62	2.67	1.05	0.24	0.23	0.35
Bred by 10 d, %	97.22	97.50	100.00	97.14	97.62	2.47	0.92	0.95	0.58
Wean-to-estrus interval, d	5.56	5.73	5.00	5.66	5.55	0.32	0.51	0.93	0.48
Subsequent total born, *n*	11.83	12.41	11.66	12.25	11.16	0.59	0.44	0.37	0.33
Litter									
Number of pigs started/sow, *n*	9.98	9.88	9.85	10.02	10.11	0.11	0.42	0.24	0.16
Number of pigs weaned/sow, *n*	9.67	9.47	9.51	9.64	9.87	0.13	0.22	0.18	0.05
Starting litter weight, kg	15.90	15.93	16.12	15.95	16.17	0.26	0.92	0.49	0.98
Weaning litter weight, kg	58.92	58.19	60.14	58.03	61.92	1.54	0.35	0.23	0.37
Piglet ADG, kg	0.257	0.260	0.271	0.250	0.268	0.06	0.10	0.52	0.91
Litter ADG, kg	2.51	2.49	2.59	2.43	2.65	0.06	0.13	0.27	0.39
Pre-wean mortality/morbidity, %	3.08	4.03	3.35	3.83	2.36	0.88	0.68	0.55	0.26

^a^Dietary treatments were as follows: 1 – 0.00, 2 – 0.075, 3 – 0.150, 4 – 0.225, 5 – 0.300% *L*-lysine HCl inclusion

^b^Dietary treatment

### Exp. 2

Sixty-six sows did not complete the study. Reasons for removal from the study included: illness resulting in significant weight loss or feed refusal, death, failure to wean more than 7 pigs, a reduced lactation period due to an early weaning event, or data entry error. Sows had an average lactation length of 20.6 ± 1.1 d. Analyzed amino acid levels differed slightly compared to the calculated levels (Table 4). The Thr:Lys and Trp:Lys ratios were higher than calculated. In addition, the Val:Lys ratio was higher than calculated as *L*-lysine HCl inclusion increased.

The average grams of SID lysine per day was not different among nor was the ME intake. Increasing *L*-lysine HCl resulted in no significant differences in sows bred by 10 d and wean to estrus interval (Table 6) and no differences were noted between dietary treatments 4 and 5. Sow feed intake was not different among the diets and averaged 5.17 kg/d. The average WEI for this study was 5.29 ± 2.1 d. In addition, subsequent total born was not influenced by dietary treatment. The average litter gain was 2.71 kg/d with no statistical differences between dietary treatment groups.

**Table 6:** Evaluation of *L*-lysine HCl on sow and litter performance for Exp. 2

	Diet^a^										
	1	2	3	4	5	SEM	Trt^b^	Parity	Trt × Parity	Linear	0.40 vs. 0.40 + Val
Sow											
Sample size, *n*	97	93	98	96	95						
Estimated SID Lys, g/d	57.5	57.0	57.3	57.0	56.7						
ADFI, kg	5.13	5.09	5.12	5.09	5.06	0.03	0.60	<0.01	0.07	0.71	0.50
Weight change, %	−4.57	−4.66	−4.09	−5.12	−5.29	0.88	0.87	<0.01	0.47	0.84	0.89
Bred by 10 d, %	96.77	95.24	95.06	94.36	99.07	1.99	0.44	<0.01	0.33	0.40	0.09
Wean-to-estrus interval, d	5.25	5.61	5.59	5.69	5.10	0.26	0.37	<0.01	0.24	0.26	0.10
Subsequent total born, *n*	13.70	13.56	13.49	13.84	13.51	0.38	0.96	0.14	0.70	0.86	0.54
Litter											
Number of pigs started/sow, *n*	11.95	11.91	12.05	12.09	11.90	0.11	0.57	0.07	0.25	0.19	0.18
Number of pigs weaned/sow, *n*	10.96	11.15	11.21	11.01	10.97	0.14	0.58	0.01	0.28	0.71	0.85
Starting litter weight, kg	21.33	21.96	21.88	22.39	22.40	0.47	0.43	0.13	0.96	0.15	0.99
Weaning litter weight, kg	71.17	73.07	71.65	72.66	71.60	1.18	0.75	<0.01	0.16	0.66	0.51
Piglet ADG, kg	0.241	0.240	0.233	0.234	0.235	0.004	0.59	<0.01	0.03	0.38	0.49
Litter ADG, kg	2.68	2.73	2.67	2.70	2.64	0.05	0.74	<0.01	0.03	0.86	0.40
Pre-wean mortality/morbidity, %	9.33	6.13	8.07	8.15	9.41	1.01	0.14	0.31	0.25	0.90	0.37

^a^Dietary treatments were as follows: 1 – 0.10, 2 – 0.20, 3 – 0.30, 4 – 0.40% *L*-lysine HCl, 5 – 0.40% *L*-lysine HCl inclusion + feed grade valine

^b^Dietary treatment

## Discussion

The levels of *L*-lysine HCl added to the diet to replace intact soybean meal were determined based on previously published literature [1]. In the literature, feeding 0.15% *L*-lysine HCl resulted in improved milk production. Therefore, the feeding of 0.15% *L*-lysine HCl became the target diet with equal increments above and below the target and including a control diet (0% *L*-lysine HCl addition). Furthermore, in Exp. 2, since no performance differences were noted in Exp. 1 at the 0.30% inclusion level, the authors determined to feed 0.10% above the 0. 30% and have a dietary treatment that included feed grade valine.

These studies demonstrate feeding feed-grade amino acids that have identical essential amino acid ratios relative to lysine for total sulfur amino acids and threonine as the corn-soybean meal based control diet resulted in similar litter growth rates. As litter weight gain was not different between dietary treatments, this indicates sows can equally and effectively utilize either intact protein or feed-grade amino acids equally for milk production.

In Exp. 1, only gilts were used in the study. The authors were concerned that a female that was still growing may have a different tolerance for feed grade amino acid inclusion levels compared to mature females. Therefore, the study was designed as a farm was first stocked and had only gilts accessible for the lactation study. This allowed for significant replication of dietary treatments with only gilts. In Exp. 2, multiparous and primiparous females were used as the herd in which Exp. 1 was conducted in had now established a mature parity structure and the authors wanted to evaluate the impact of feed grade amino acids on mature females. Furthermore, gilts were used in this study to be able to have a comparison across both studies. The outcomes were not different between the two experiments.

Data from Srichana et al. [8] demonstrated that the gilt SID g lysine/d requirement during lactation should be approximately 60-64 g/d based on approximately 2.35 kg/d of litter growth rate. When gilts are fed below this level, piglet ADG declines from 288 g/d (63 g SID lysine/d) to 262 g/d (48 g SID lysine/d). If 10 piglets are nursed for 20 d of lactation, this would result in a reduced litter growth rate of 5.2 kg of total litter growth or approximately 0.52 kg/pig at the time of weaning. Based on this information, it was estimated that the gilts would need to consume roughly 25 g SID lysine/kg litter growth rate (LGR) for optimal piglet weight gain. Based on the average consumption values, the gilts consumed approximately 25.3 g SID lysine/kg LGR or 64 g SID lysine/d, which was in the SID lysine range estimated by Srichana to optimize litter growth rate. In Exp. 2, sow feed intake was slightly lower than anticipated to allow for the SID lysine not to be overfed to further evaluate the SID valine requirement, which resulted in approximately 21.3 g SID lysine/kg LGR. Furthermore, feed intake in Exp. 1 resulted in gilt weight gain during lactation and not body weight loss.

Increasing feed-grade lysine to 0.30% with supplemental threonine and methionine resulted in 8.8% decrease in soybean meal and 8.1% increase in corn (Table 1). This shift in soybean meal resulted in a decrease of many amino acids, which potentially could result in a creating a limiting amino acid ratio relative to lysine. The substitution of the soybean meal with feed-grade amino acids did not alter performance. Since there were no differences in performance noted, the results indicate that all essential and non-essential amino acids were adequate and were at or above the NRC (2012) recommendation [9]. These data support the work of Cooper et al. [3] who also demonstrated feeding feed-grade amino acids in lactation diets will not impact sow or litter performance.

Total valine intake, in Exp. 1, was 46 g/d total valine in dietary treatment 5; therefore, valine likely was not a limiting amino acid. In Exp. 2, at the highest level of *L*-lysine HCl inclusion (dietary treatment 4), total valine intake was estimated at 36.6 g/d. Based on this calculation, total valine would likely be a limiting amino acid according to NRC (2012) [9]. A sow with a 2.7 kg/d litter gain during lactation would require approximately 48.9 g total valine/d and a gilt with a 2.5 kg/d litter gain would require approximately 45.7 g total valine/d. Dietary analysis in Exp. 2 for total valine did not appear accurate. The total valine levels were expected to decline significantly; however, the values appeared relatively equal across the dietary treatments. This was unexpected as the other amino acid levels relative to lysine either decreased the expected level from dietary treatment 1 to dietary treatment 4 or were close to the calculated level. Therefore, the authors chose to use the calculated values rather than the analyzed values when determining the amino acid ratio response.

In Exp. 2, estimated calculated total valine intake ranged from 52.5 g to 43.4 g/d (SID Val:Lys ratio ranging from 78 to 64%) in the first four dietary treatments as feed grade lysine increased. In the fifth dietary treatment, the addition of valine would have increased the calculated total valine intake to 45.8 g/d (SID Val:Lys ratio of 69%). Carter et al. [10] reported increasing the dietary Val:Lys ratio from 76 to 122% did not affect any sow or litter performance trait. Richert et al. [11] reported in order to maximize litter growth rate, the diet should contain a total Val:Lys ratio of 120%. In order to achieve this ratio in most practical lactation diets, feed-grade valine would need to be added to the diet. However, in research conducted by Boyd et al. [12] and Gaines et al. [13], the necessary total Val:Lys ratio was determined to be between 73 and 86%. Although the addition of valine was not at a level high enough to be between 73 and 86%, the supplemental valine to a diet that was below the requirement should have resulted in improved litter growth rate, which it did not.

In Exp. 2, sows lost weight in response to the controlled feed allowance and consumed on average 64.0 g total lysine/d. Although sows lost body weight during lactation, sow performance was not altered with the increase in feed-grade amino acids. The quadratic response of subsequent total born mimics the reduction of daily feed intake and is likely a result of the low feed intake on the total born rather than an associated with a specific dietary treatment. Furthermore, it was demonstrated when feed-grade lysine was included at 0.40%, the calculated SID Val:Lys ratio was 64%, which did not impact performance. It is unknown if feeding additional valine to increase the level to a minimum of 73% would have resulted in an improvement in litter growth rate.

Tryptophan and isoleucine ratios in Exp. 1 were close to and above the NRC (2012) recommendation [9]. In Exp. 2, the SID Trp:Lys ratio declined to slightly under the NRC (2012) recommendations [9]. Current NRC (2012) [9] recommendations for the SID Trp:Lys ratio would be at 19%. In addition, Exp. 2 would also have been feeding slightly below the NRC (2012) recommendation for SID M + Cys:Lys. Based on this information, the findings may be different if these ratios were increased. However, in Exp. 2, ADFI does not appear to be influenced by the declining SID Trp:Lys levels.

## Conclusion

In conclusion, feed-grade lysine can be added to corn-soybean meal based lactation diets at a 0.40% inclusion rate with supplemental methionine and threonine without altering reproductive or lactation performance. In addition, replacing intact protein with feed-grade amino acids in lactation diets does not have negative effects on performance provided amino amino acid ratios to lysine of SID M + C (49%), SID Thr (65%), SID Trp (16%), and SID Val (64%) are maintained.

## Abbreviations

ADFI: Average daily feed intake
ADG: Average daily gain
BW: Body weight
C/Cys: Cysteine
CP: Crude protein
Cu: Copper
d: day(s)
Exp: Experiment
Fe: Iron
HCl: Hydrochloride
HMTBa: 88% 2-hydroxy, 4-methylthio butanoic acid
I: Iodine
kg: kilogram(s)
LGR: Litter growth rate
Lys: Lysine
M/Met: Methionine
ME: Metabolizable energy
Mn: Manganese
MOD: Modified
Se: Selenium
SID: Standard illeal digestible
Thr: Threonine
Trp: Tryptophan
Val: Valine
WEI: Wean to estrus interval
Zn: Zinc
